# Neuroanatomical Alterations in Tinnitus Assessed with Magnetic Resonance Imaging

**DOI:** 10.3389/fnagi.2016.00221

**Published:** 2016-09-21

**Authors:** Thomas W. Allan, Julien Besle, Dave R. M. Langers, Jeff Davies, Deborah A. Hall, Alan R. Palmer, Peyman Adjamian

**Affiliations:** ^1^Medical Research Council Institute of Hearing Research, The University of NottinghamNottingham, UK; ^2^Nottingham Hearing Biomedical Research Unit, National Institute for Health Research (NIHR)Nottingham, UK; ^3^Otology and Hearing Group, Division of Clinical Neuroscience, School of Medicine, The University of NottinghamNottingham, UK

**Keywords:** tinnitus, brain anatomy, auditory cortex, voxel-based morphometry, surface-based morphometry

## Abstract

Previous studies of anatomical changes associated with tinnitus have provided inconsistent results, with some showing significant cortical and subcortical changes, while others have found effects due to hearing loss, but not tinnitus. In this study, we examined changes in brain anatomy associated with tinnitus using anatomical scans from 128 participants with tinnitus and hearing loss, tinnitus with clinically normal hearing, and non-tinnitus controls with clinically normal hearing. The groups were matched for hearing loss, age and gender. We employed voxel- and surface-based morphometry (SBM) to investigate gray and white matter volume and thickness within regions-of-interest (ROI) that were based on the results of previous studies. The largest overall effects were found for age, gender, and hearing loss. With regard to tinnitus, analysis of ROI revealed numerous small increases and decreases in gray matter and thickness between tinnitus and non-tinnitus controls, in both cortical and subcortical structures. For whole brain analysis, the main tinnitus-related significant clusters were found outside sensory auditory structures. These include a decrease in cortical thickness for the tinnitus group compared to controls in the left superior frontal gyrus (SFG), and a decrease in cortical volume with hearing loss in left Heschl’s gyrus (HG). For masked analysis, we found a decrease in gray matter volume in the right Heschle’s gyrus for the tinnitus group compared to the controls. We found no changes in the subcallosal region as reported in some previous studies. Overall, while some of the morphological differences observed in this study are similar to previously published findings, others are entirely different or even contradict previous results. We highlight other discrepancies among previous results and the increasing need for a more precise subtyping of the condition.

## Introduction

Tinnitus, the perception of a phantom sound in the absence of an external source, is experienced chronically by approximately 5–15% of the population (Baguley et al., 2013). The onset of tinnitus is typically associated with aging or exposure to loud noise, cumulative or sudden, such that hair cell damage occurs with subsequent hearing impairment. Tinnitus can cause significant distress, causing problems such as depression, anxiety and sleep disorders (Henry et al., 2005).

The exact cause of tinnitus and its associated pathophysiology remains unknown. Although tinnitus is commonly initiated by damage to the peripheral auditory system, it is believed that the sound percept is generated and maintained in the brain. This has been confirmed by surgical interventions where the auditory nerve is bisected yet the perception of the tinnitus sound remains (House and Brackmann, 1981). Current consensus is that the initial cause of many forms of tinnitus is strongly related to cochlear damage and the resulting hearing loss, which may cause changes to neural coding properties (Seki and Eggermont, 2003).

Recently, both functional and structural imaging have been used to investigate changes in the brain associated with tinnitus. Functional studies based on changes in regional cerebral blood flow (using positron emission tomography; PET) have found regions of the brain associated with the perception of the tinnitus sound (Mirz et al., 1999, 2000a,b; Reyes et al., 2002). However, PET has limitations due to the required administration of radioactive tracer restricting longitudinal studies. Functional magnetic resonance imaging (fMRI) has also been used to measure changes in brain activity in people with tinnitus. This method has high spatial resolution, but significant acoustic noise is generated by the scanner during the imaging process that may have a confounding effect on the results (Melcher et al., 2000; Lanting et al., 2008, 2009). Moreover, fMRI is able to detect persistent changes in neural baseline activity that can be associated with chronic tinnitus. Magneto- and electroencephalography (MEG, EEG) have been used to examine differences in power spectra and to localize regions of the brain associated with tinnitus. However, these methods suffer from poor spatial resolution and results are contradictory (for review see Adjamian et al., 2009). All functional imaging methods have confounding issues that relate to task performance, and how well the participant is able to describe the changes they are experiencing.

Anatomical brain changes are believed to arise from plasticity and reorganization of the brain. Analysis of high resolution MRI anatomical data of the brain, thus complements functional imaging. Various studies have shown that functional changes are directly linked to structural changes in tinnitus (Mühlau et al., 2006; Schneider et al., 2009; Husain et al., 2011; Leaver et al., 2011, 2012; Mahoney et al., 2011; Aldhafeeri et al., 2012; Schecklmann et al., 2012; Boyen et al., 2013; Melcher et al., 2013). Adjamian et al. (2014) recently provided a comprehensive review of this literature.

Changes in structure, gray and white matter volumes and brain shape are indicative of differences in prolonged neuronal activity and connectivity between brain regions (Pfefferbaum et al., 1994; Good et al., 2001; Draganski et al., 2004; Maguire et al., 2006). We have previously reviewed several approaches to structural analysis that have been growing in popularity in recent years, including: voxel-based morphometry (VBM), surface-based morphometry (SBM), deformation-based morphometry (DBM), tensor-based morphometry (TBM) and diffusion tensor imaging (DTI) (Adjamian et al., 2014). VBM allows assessment using statistical metrics of voxel-wise changes in the gray matter volume of the neocortex between populations, or in any given population relative to a clinical measure. However, VBM has been criticized for being sensitive to image registration procedures that can yield spurious results (Bookstein, 2001). On the other hand, using the surface of the brain, SBM highlights the cortical folding of the brain and avoids the registration problems, to some extent, by investigating differences in the area, thickness of tissue or the curvature of the cortex between subjects (Winkler et al., 2010).

Current evidence regarding the tinnitus-related structural changes in the brain has produced a range of contradictory and varied results. Mühlau et al. (2006) were the first to show structural changes related to tinnitus using whole brain and region-of-interest (ROI) voxel-wise VBM analyses. This study showed a reduction in gray matter in subcallosal areas, such as the nucleus accumbens (NAc), and an increase in the medial geniculate nucleus (MGN). However, other studies have failed to replicate these results using largely similar methods (e.g., Landgrebe et al., 2009; Husain et al., 2011; Melcher et al., 2013). In line with Mühlau et al. (2006) findings, Rauschecker et al. (2010) suggested a gating model in which tinnitus results from a failure to inhibit noise, allowing unpleasant noise signals to reach the auditory cortex (AC). This model is based upon evidence from human neuroimaging and animal studies and involves cortical and subcortical regions consisting of the amygdala, the NAc, the ventromedial prefrontal cortex (vmPFC), and the reticular nucleus of the thalamus (Leaver et al., 2011, 2012; Seydell-Greenwald et al., 2014). This limbic corticostriatal pathway has been shown to play an important role in the suppression of unpleasant sounds. Consequently, abnormalities within these areas of the brain may lead to the perception of a tinnitus sound and the negative emotions associated with chronic tinnitus. As this model predicts tinnitus-related changes in the activity of specific structures, it can be evaluated using MRI-based morphological analysis techniques.

In a recent article, we identified various factors which may underlie the reported inconsistent findings (Adjamian et al., 2014). These include the heterogeneity of tinnitus characteristics such as its etiology, duration and lateralization. Moreover, in most studies, important parameters that may independently affect brain anatomy have not been adequately controlled for, such as age and hearing loss (Lee et al., 2007; Crippa et al., 2010). Another important factor may be the small size of participant groups, often due to recruitment difficulties, with sometimes as few as 11 tinnitus participants. Small sample sizes result in low statistical power, such that effects from one or two participants can dramatically change the overall outcomes. In addition to the low statistical power, many studies employ thresholds uncorrected for multiple comparisons, which increases the chance of false positives. Another possible reason for the inconsistent findings might be the masks used to specify ROIs in the analysis. Six groups have used the same masks as defined in Mühlau et al. (2006), but with varying results (Mühlau et al., 2006; Landgrebe et al., 2009; Husain et al., 2011; Leaver et al., 2011; Boyen et al., 2013; Melcher et al., 2013). Others have focused upon whole-brain analysis (Schecklmann et al., 2012, 2013), specific ROIs such as Heschl’s gyri (Schneider et al., 2009) and the inferior/superior/middle frontal gyri (Aldhafeeri et al., 2012), large ROIs such as the temporal lobe, upper brain stem and bilateral orbito-frontal cortices (Mahoney et al., 2011), or a range of brain regions such as the thalamus, caudate, putamen and globus pallidus (Leaver et al., 2012). This range of foci means that it is difficult to distinguish a consistent pattern across studies.

Given the previous inconsistent findings, we aimed to address some of these methodological issues using MRI data collected from a large cohort of tinnitus participants and matched controls at partner research centers in Nottingham. We tested the hypothesis that tinnitus is accompanied by changes in gray and white matter compared to non-tinnitus controls. More specifically, we aimed to detect structural changes using VBM and SBM, whilst controlling for variables such as tinnitus severity, hearing loss, and age. Based on the gating mechanism proposed by Rauschecker et al. (2010), we hypothesize that these changes in brain anatomy will be located in the MGN, the vmPFC, and the NAc. We use various ROI masks based on previous studies, including those by Mühlau et al. (2006) and Leaver et al. (2011) to examine areas beyond the subcallosal region. Finally, given that tinnitus is an ongoing sensation that has been shown to affect resting state activity (Husain and Schmidt, 2014), we also investigated anatomical changes in the areas constituting the default mode network (DMN), which is linked to resting-state activity (Greicius et al., 2003).

## Materials and Methods

### Subject Recruitment

One hundred twenty eight participants (73 tinnitus and 55 controls) were recruited as part of other functional imaging studies at the Medical Research Council (MRC) Institute of Hearing Research (IHR; *n* = 61) and the National Institute for Health Research (NIHR) Nottingham Hearing Biomedical Research Unit (BRU; *n* = 67). The IHR cohort had been recruited as part of MEG studies to investigate oscillatory and evoked responses in tinnitus (Adjamian et al., 2012; Sereda et al., 2013). The BRU cohort was recruited for a study examining hearing aid benefits for tinnitus with functional MRI (Davies et al., 2014). The IHR study was approved by the National Health Service (NHS) East Midlands Nottingham local research ethics Committee 2, and sponsored by the MRC. The BRU study was approved by the North Nottinghamshire Research Ethics Committee, and sponsored by the NHS Nottingham University Hospital Trust.

Audiograms comprising frequencies from 0.25 to 12 kHz were acquired for each subject. Clinically normal hearing was based on the average of the pure-tone hearing threshold levels 250, 500, 1000, 2000 and 4000 Hz <20 dB Hearing Loss (HL; British Society of Audiology, 2011). Participants with tinnitus completed either the Tinnitus Handicap Inventory (THI; Newman et al., 1996; *N* = 30, the IHR cohort) or Tinnitus Handicap Questionnaire (THQ; Kuk et al., 1990; *N* = 43, the BRU cohort) to assess its severity. Because these questionnaire scores have the same range (0–100) and show high convergent validity (Fackrell et al., 2014), we applied a simple stratification to combine both sets of scores so that all tinnitus participants had a tinnitus severity score that fell within one of five categories: grade one, 0–16 (low); grade two, 17–36 (mild); grade three, 37–56 (moderate); grade four, 57–76 (severe); or, grade five, 77–100 (catastrophic). These boundaries were informed by a UK THI grading (McCombe et al., 2001).

### Group Classifications

Three separate groups of participants were defined for statistical analysis. The aim of these groups is to isolate particular features so that we could maximize the statistical power to detect various potential effects.

#### Group 1—All Subjects

The first group consisted of all participants in the study, divided into two subgroups of tinnitus participants and non-tinnitus controls. This comparison maximized the statistical power available to detect possible changes related to tinnitus.

#### Group 2—Severe Tinnitus vs. Matched Controls

The second group consisted of participants with severe or catastrophic tinnitus and controls individually matched for age, gender and hearing loss. This comparison was made to assess the effect of highly intrusive tinnitus compared to a matched sample.

#### Group 3—Tinnitus With Clinically Normal Hearing vs. Matched Controls

The final group consisted of tinnitus participants with clinically normal hearing, again matched individually for age and gender to controls with clinically normal hearing. This comparison isolated the effect of tinnitus from the effect of hearing loss.

**Figure 1:**
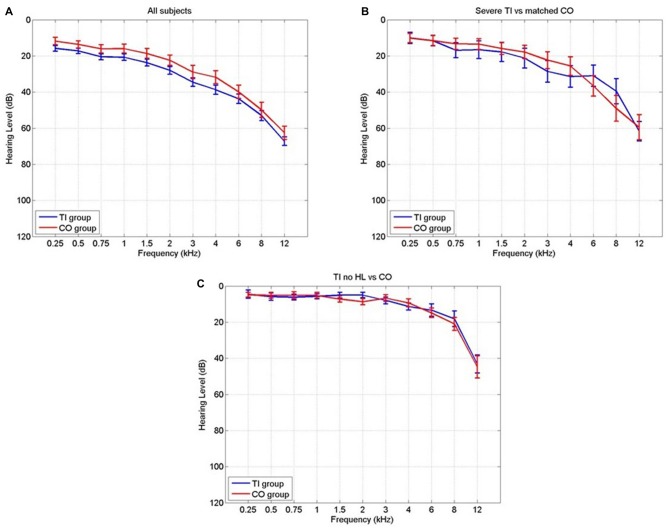
**The audiograms for each comparison group for the tinnitus participants (blue) and the controls (red) in (A) all subjects, (B) severe tinnitus and matched controls and (C) tinnitus with no hearing loss and matched controls**.

Information and number of participants pertaining to each of the subgroups are listed in Table 1 and their mean audiograms are shown in Figures 1A–C. There were no significant differences in age, gender or hearing loss between the tinnitus participants and control participants in any of the three groups.

**Table 1:** **Demographic information and hearing status for each of the three groupings**.

Group	Subgroup	*N*	Age (years)				Gender		PTA (dB HL)
			Mean	Std	Min	Max	Male	Female	Mean (both ears)
1 (All)	Tinnitus	73	58.38	12.41	24	80	43	30	28.62
	Controls	55	56.91	16.39	19	76	30	25	24.19
2 (Severe Tinnitus vs. matched Controls)	Tinnitus	16	55.06	15.53	24	80	8	8	21.51
	Controls	16	53.69	14.85	25	72	8	8	20.91
3 (Tinnitus with clinically normal hearing vs. matched controls)	Tinnitus	15	47.60	16.66	24	80	6	9	8.22
	Controls	15	50.20	17.25	20	71	6	9	8.62

### Data Acquisition

Subjects were scanned either on a 3-T or 1.5-T Philips scanner by means of a high resolution magnetization-prepared rapid gradient-echo (MPRAGE) acquisition (resolution = 1 × 1 × 1 mm^3^, repetition time (TR) = 8 s, echo time (TE) = 3.74 ms, field of view (FOV) = 256 × 256 × 160 mm^3^). One hundred twenty one participants were scanned on the 3 T scanner, and 7 (4 tinnitus and 3 non-tinnitus controls) on the 1.5 T, because, the 3 T scanner was unavailable.

#### VBM Data Processing

The data processing was performed using Statistical Parametric Mapping (SPM8^1^). Each participant’s anatomical image was segmented into gray matter, white matter, cerebrospinal fluid (CSF) and other tissues. For each group (described above), Diffeomorphic Anatomical Registration Through Exponentiated Lie algebra (DARTEL: Ashburner, 2007) was used to create a white matter and gray matter template using segmented images of participants from all of the groups to improve the registration to a common space. That is, each group had its own gray and white matter template to which both the tinnitus participants and controls were realigned. Whilst aligning the individual images to the template, the volume of white and gray matter was preserved (i.e., modulated images were computed).

The data were then resampled to 2-mm isotropic resolution, aligned with Montreal Neurological Institute (MNI) space and subsequently smoothed with an isotropic 10-mm full-width at half-maximum (FWHM) kernel. Finally, these images were set at a threshold of 5% to compensate for edge voxels and the blurring effect caused by smoothing the data.

Statistical maps were produced using linear regression through an ANCOVA model with a group factor (tinnitus or control) and additional covariates for tinnitus severity grading (1–5); controls were all assigned a value equal to the average of the tinnitus subgroup in order for this regressor to be orthogonal to the group factor), left and right ear pure tone averages (PTA) over the tested frequencies to 8 kHz, age, and gender. The group factor and all four covariates were tested for statistical significance. In addition, for each subject, the whole brain gray matter and white matter volumes were used as an additional covariate in the respective ANCOVA models to compensate for whole brain volume differences between subjects. In an alternative model, the raw THI/THQ scores were used as a covariate instead of the tinnitus severity grade, but the results were equivalent and are therefore not shown here. All the statistical maps were family-wise error (FWE) corrected for multiple comparisons using Gaussian Field theory with a confidence threshold of 0.05. The statistical analysis was run on the whole brain as well as restricted to each of the masks defined below.

#### FreeSurfer Data Processing

Cortical surface reconstruction was performed using the standard FreeSurfer v 5.3.0^2^ pipeline run on a Linux CentOS 6 platform. In brief, this includes removal of non-brain tissues, automated Talairach transformation, segmentation of the subcortical white matter and deep gray matter volumetric structures, intensity normalization, tessellation of the gray and white matter boundary, automated topological correction and surface deformation. The technical details are fully described in prior publications (Dale et al., 1999; Fischl et al., 1999a,b; Fischl and Dale, 2000).

Measures of each subject’s cortical thickness, area and volume were computed. Each subject’s cortical surfaces were morphed onto the standard inflated brain and the thickness, area and volume values were smoothed with a 10-mm FWHM Gaussian kernel. The same ANCOVA model as in the VBM analysis, with one group factor and four covariates, was fitted to each measure (one difference with the VBM analysis however is that we did not use mean/total thickness/area/volume as an additional covariate). Monte-Carlo simulations were run to correct for multiple comparisons at the cluster level (Hayasaka and Nichols, 2003), as implemented in Freesurfer (Hagler et al., 2006). For the simulations, the voxelwise (uncorrected) threshold was set to *p* = 0.01 and the clusterwise (corrected) threshold was set to *p* = 0.05. Statistical analysis was conducted on the whole surface as well as in each of the masks, defined below.

### Masks

To complement the whole-brain analyses, 10 masks were defined to assess changes in particular regions of the brain that might be attributable to the tinnitus percept. These masks were chosen based on the regions specified by the gating mechanism proposed by Rauschecker et al. (2010), and those used by Mühlau et al. (2006) that had shown tinnitus-related changes in brain volume.

**Figure 2:**
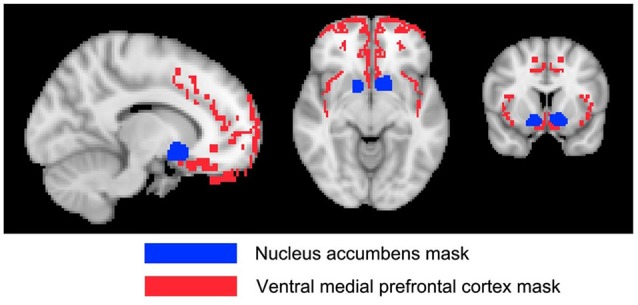
**The Rauschecker model brain regions [nucleus accumbens (NAc) and ventromedial prefrontal cortex (vmPFC)] that have been defined as a mask for further analysis**.

Detailed definition of each mask is given in Table 2. The masks based on Rauschecker’s gating mechanism are shown in Figure 2. The WFUpickatlas in SPM8 was used to define the masks. The AC mask (Brodmann areas 22, 41 and 42) was dilated by 2 mm in all directions to accommodate small registration errors and individual variability issues. The NAc mask was defined from the WFUpickatlas, and the vmPFC consisted of Brodmann areas 10, 11, 12, 13, 14, 25 and 32. Heschl’s gyrus (HG; Brodmann area 41) was also dilated by 2 mm to account for small misregistration effects. The DMN comprised the posterior cingulate cortex (pCC), medial frontal gyrus (mFC) and middle temporal gyrus (MTG). The superior temporal gyrus (STG) was selected as it relates to normal auditory functioning, but does not include all areas of the AC that lie on the supratemporal plane. The masks for the AC, HG and STG had overlapping regions. The vmPFC and DMN also showed overlap.

**Table 2:** **The definitions of the masks used and how they were defined**.

Mask	Brodmann areas	WFUpickatlas	MNI coordinates (*x, y, z*)	Volume (bilateral, cm^3^)
Cochlear nucleus (CN)	–	–	±10, −38, −45	1.30
Superior olivary complex (SOC)	–	–	±13, −35, −41	1.30
Inferior colliculus (IC)	–	–	±6, −33, −11	1.30
Medial geniculate nucleus (MGN)	–	–	±17, −24, −2	4.11
Heschl gyrus (HG)	41	–	–	13.45*
Auditory cortex (AC)	22, 41 and 42	–	–	74.74*
Superior temporal gyrus (STG)	–	Superior temporal gyrus	–	83.97
Nucleus accumbens (Na_C_)	–	Nucleus accumbens left and right	–	4.25
Ventromedial prefrontal cortex (vmPFC)	10, 11, 12, 13, 14, 25 and 32	–	–	77.19
Default mode network (DMN)	–	Posterior cingulate, medial frontal gyrus and middle temporal gyrus	–	120.90

*Indicates volume after 2 mm dilation;—Indicates no information available.

**Figure 3:**
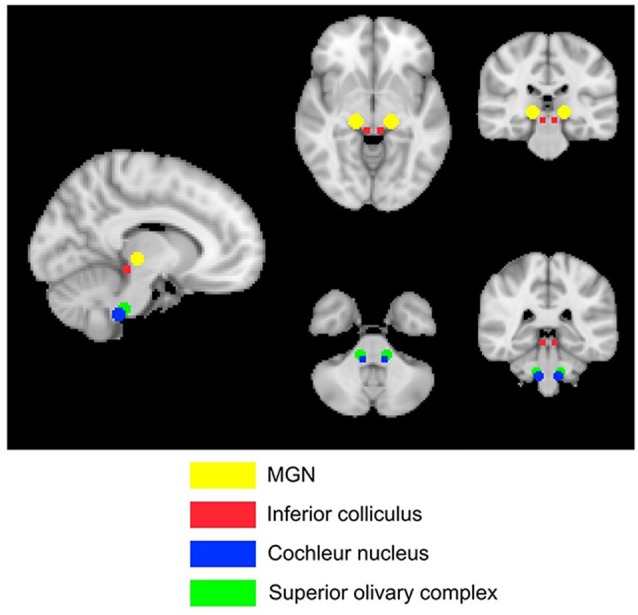
**The Mulhau masks that have previously shown anatomical changes relating to tinnitus**.

Four subcortical masks were defined using coordinates based on Mühlau’s study, to determine if their results could be replicated with our larger cohort of participants. These masks are shown in Figure 3. Bilateral 5-mm radius spheres were defined for the Cochlear nucleus (CN), the Superior olivary complex (SOC), and the Inferior colliculus (IC). In addition to these, bilateral 8 mm radius spheres were defined in the MGN.

All of the masks were used for the VBM analysis. Five of these masks were used for the SBM analysis (AC, DMN, HG, vmPFC and STG) as the other masks targeted subcortical structures. To create the surface masks, the volumetric masks were projected onto the standard inflated brain and manually corrected to remove non-contiguous vertices due to projection errors (corresponding to voxels contiguous in volumetric space but belonging to non-contiguous gyri).

### ROI Analysis

For all of the masks and groups, ROI analyses were performed to compute the magnitude of differences between the tinnitus participants and controls. The various measures of interest (i.e., gray and white matter volume for VBM; surface thickness, area, and volume for SBM) were averaged (gray and white matter volume, surface cortical thickness) or summed (surface area and volume) across the voxels or vertices in a mask, and statistically compared between subgroups using *t*-tests. In Freesurfer, surface area/volume measures were normalized by the total surface area/volume of each hemisphere and all individual measures were averaged between the left and right surface masks.

## Results

### Correlation Analysis

Pearson correlation coefficients were calculated between hearing loss PTA, age and tinnitus severity data to assess whether any covariates were interdependent.

**Figure 4:**
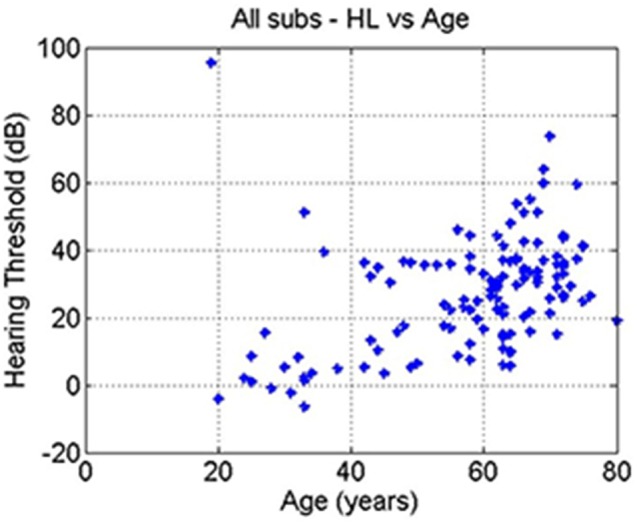
**The age of each subject plotted against their hearing threshold**.

The correlation between age and hearing loss across all subjects was significant (*r* = 0.41, *p* = 10–6; Figure 4). The correlations between tinnitus severity and hearing loss (*r* = 0.08, *p* = 0.36) or tinnitus severity and age (*r* = −0.00, *p* = 0.98) did not reach significance.

### Morphometry

Overall, we found a number of clusters of significant effects in various masks and for different contrasts, varying in size. A summary of the significant effects of interest (Tinnitus vs. Controls and Tinnitus severity) is given in Table 3 and they are described in detail in the following sections and shown in Figures 6–11. Full details of all findings, including significant effects of other covariates, are presented in the Supplementary Material Tables SI1–SI5.

**Table 3:** **Summary of results**.

VBM			
	Gray matter		White matter
Grouping 1. (All TI vs. All controls)	Reduction for TI vs. CO in right HG (0.008 cm^3^) and with increasing TI severity in right DMN (0.056 cm^3^).		Reduction in TI vs. CO in right MGN (0.50 cm^3^)
	ROI analysis: increase in SOC for TI vs. CO (5.5%)	
Grouping 2. (Severe TI vs. Matched controls)	No Differences		Reduction in TI vs. CO in right MGN (0.19 cm^3^)
Grouping 3. (TI with Clinically normal hearing vs. matched controls)	No Differences		Increase in TI vs. CO in left HG (0.008 cm^3^); Increase with TI severity in left CN (0.008 cm^3^)

**SBM**			

	**Thickness**	**Area**	**Volume**

Grouping 1. (All TI vs. All controls)	Decrease for TI vs. CO in left AC/STG (4.51 cm^2^), left superior frontal gyrus (5.04 cm^2^), right STS (1.69 cm^2^) and right HG (0.33 cm^2^). Increase with TI severity in right middle Temporal gyrus (2.61 cm^2^), and right rostro medial Frontal gyrus (2.02 cm^2^).	Decrease with TI severity in right precuneus (12.5 cm^2^)	Decrease for TI vs. CO in right HG (0.27 cm^2^) and with TI severity in right precuneus (8.35 cm^2^)
	ROI analysis: 2.1% decrease for TI vs. CO in AC	ROI analysis: increase for TI vs. CO in vmPFC (1.31%) and decrease in AC (−2.1%)	ROI analysis: decrease for TI vs. CO in Auditory cortex (−3.9%), HG (−4.5%) and STG (−2.4%)
Grouping 2. (Severe TI vs. Matched controls)	Decrease for TI vs. CO in left AC (0.85 cm^2^).	No Differences	No Differences
Grouping 3. (TI with Clinically normal hearing vs. matched controls)	Decrease for TI vs. CO in AC/STG (1.17 cm^2^) and right rostro-middle frontal gyrus (1.71 cm^2^). Increase with tinnitus severity in left midtemporal gyrus (3.18 cm^2^)	Decrease with TI severity in left HG/AC (3.18 cm^2^), right superior parietal gyrus (5.80 cm^2^) and right posterior cingulate (6.37 cm^2^)	Decrease with TI severity in left HG/AC (2.50 cm^2^)

*Results of VBM voxelwise analysis and SBM vertexwise analysis are reported as volume and surface area of significant clusters. ROI analysis results are reported in % change relative to the average/total ROI value averaged across TI and CO groups (TI, Tinnitus; CO, Control; MTG, Middle Temporal Gyrus; STS, Superior Temporal Sulcus. Other abbreviations as in Table 1)*.

#### Grouping 1—All subjects

##### Whole-head voxel/vertexwise analysis

In both the VBM and SBM analyses, by far the largest effects (in terms of extent of the significant clusters) were for the age and gender covariates. Regarding tinnitus, tinnitus severity, and hearing loss, only a few small clusters were found and only when using SBM.

**Figure 5:**
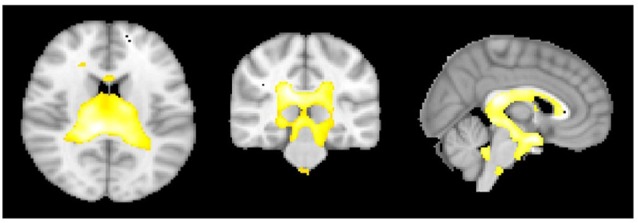
**The areas showing that as age increases there is a significant increase in gray matter volume at the cerebrospinal fluid (CSF) boundaries for the regression group of all subjects using age as the regressor of interest**.

Whole brain VBM analysis (see Tables SI1,SI2) revealed many significant clusters in various parts of the brain, corresponding mainly to an increase in gray matter volume with age (totalling 10,234 voxels or 81.9 cm^3^), and to a lesser extent a decrease in white matter volume with age (totalling 34.7 cm^3^) and larger gray matter volume for males vs. female (totalling 13.9 cm^3^). The largest of these clusters was an increase in gray matter volume with age in a 20 cm^3^ volume near the boundaries of the ventricles (Figure 5). This cluster contains very little actual gray matter, being predominantly white matter and CSF. So the voxel grayscale is likely contaminated. As the ventricles are known to expand during the ageing process due to cortical atrophy, there is a likely explanation for this finding and so it is not discussed further. Other significant clusters were small in extent (<1 cm^3^) and there were no significant effects of tinnitus, tinnitus severity or hearing loss.

**Figure 6:**
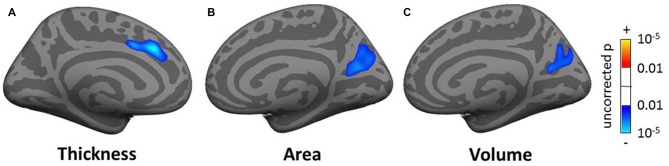
**Clusters showing a significant effect (*p* < 0.05 family wise error (FWE)-corrected) of tinnitus in the superior frontal gyrus (SFG; A) and of tinnitus severity in the pre-cuneus (B,C) in the whole-brain surface-based morphometry (SBM) analysis.** Blue areas correspond to a negative effect (decrease in thickness for the tinnitus group vs. the control group and decrease in area and volume for increasing tinnitus severity).

In the whole brain SBM analysis (see Tables SI3–SI5), various clusters showed a decrease of cortical thickness, area and/or volume with age (total areas of the clusters were 558.5, 224.2 and 610.0 cm^2^ respectively). There was an increase in cortical area and/or volume for males compared to females (total cluster areas were 1205.1 and 836.3 cm^2^ respectively). Additionally, there were clusters of decreased cortical thickness for males in the left hemisphere (total area 7.9 cm^2^). There were smaller significant clusters for tinnitus, tinnitus severity and hearing loss. These included a decrease in cortical thickness for the tinnitus group compared to controls in a 5.0 cm^2^ area in the left superior frontal gyrus (SFG; Figure 6A), a decrease in cortical area and volume with tinnitus severity in an area of the right precuneus (over 12.5 and 8.4 cm^2^ respectively; Figures 6B,C), a decrease in cortical volume with hearing loss in left HG (over 4.8 cm^2^; not shown) and a decrease in cortical area and volume with hearing loss in the right fusiform gyrus (over 6.2 and 5.3 cm^2^ respectively; not shown).

##### Masked voxel/vertexwise analysis

When restricting the VBM and SBM analyses to the various masks, the majority of clusters of significance were again found for the effects of age and gender and these effects were generally in the same direction as in the whole-head analysis. These clusters will not be detailed here (see Tables SI1–SI5). There were additional clusters of significance for tinnitus, tinnitus severity and hearing loss, which will be summarized below.

**Figure 7:**
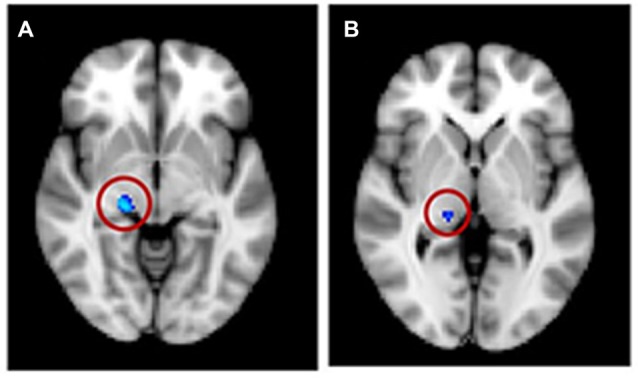
**Significant voxels (circled) found in the medial geniculate nucleus (MGN) for a reduction in white matter for tinnitus participants compared to controls for (A) regression group 1, all subjects and (B) regression group 2, severe tinnitus matched to controls**.

For the VBM masked analyses, we found a decrease in gray matter volume in the right HG (1 voxel or 0.008 cm^3^; not shown) and in white matter volume for the tinnitus group compared to the control group in the right MGN (63 voxels or 0.50 cm^3^; Figure 7A), a decrease in gray matter volume in the right DMN (0.056 cm^3^) with increasing tinnitus severity (not shown). There were also two clusters of decreasing white matter volume with hearing loss in the left HG (found using AC, HG and STG masks) and left vmPFC. We also found one cluster of increasing white matter volume with hearing loss in the left NAc (all clusters <0.6 cm^3^; not shown).

**Figure 8:**
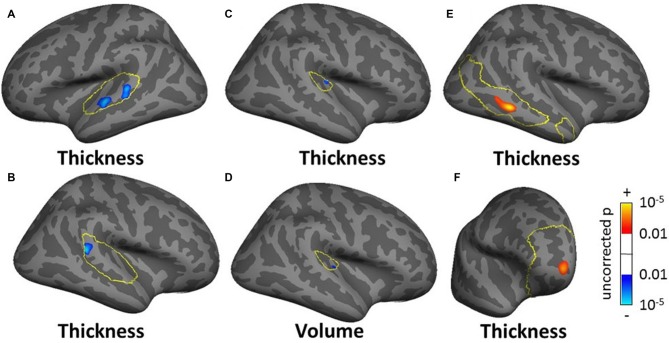
**Clusters showing a significant effect (*p* < 0.05 FWE-corrected) of tinnitus in left AC (A) right superior temporal sulcus (STS; B) right Heschl’s gyrus (HG) (C,D) and of tinnitus severity in right middle temporal gyrus (MTG) and PFC (E,F) in the masked SBM analysis.** The yellow outlines depict the masks used to restrict the vertexwise analysis (auditory cortex, AC for **A,B** HG for **C,D** default mode network (DMN) for **E**; PFC for **F)**. Blue areas correspond to a negative effect (decrease in thickness or volume for the tinnitus group vs. the control group) and the red areas to a positive effect (increasing thickness for increasing tinnitus severity).

For the SBM masked analyses, we found several clusters of decreased cortical thickness or volume for the tinnitus group compared to controls. These included, two clusters of decreased thickness in the left AC (total cluster area 4.51 cm^2^, Figure 8A, found using both the AC and STG masks), one cluster of decreased thickness in the bank of the right superior temporal sulcus (STS; 1.69 cm^2^, Figure 8B, found using both the AC and STG masks), one cluster of decreased thickness and one of decreased volume in right HG (0.33 and 0.27 cm^2^ respectively, Figures 8C,D) and one cluster of decreased thickness in the left SFG, identical to the one found in the whole brain analysis (Figure 6A). There were also two clusters of increasing cortical thickness with increasing tinnitus severity: one in the right MTG (2.61 cm^2^, Figure 8E) and one in the right rostro-medial frontal gyrus (2.02 cm^2^, Figure 8F).

##### ROI analysis

In the ROI analysis, we averaged the various measures across all voxels/vertices of each mask bilaterally and compared them between the tinnitus and control groups. This analysis showed additional significant effects, some of which were similar to the voxel/vertexwise effects. The only significant effect for the VBM ROI analysis was a 5.5% increase in gray matter volume for the tinnitus group in the SOC. For the SBM ROI analysis, we found a 2.1% decrease in cortical thickness in AC (similar to the vertex-wise effect shown in Figures 8A,B), a 1.3% increase in cortical area in vmPFC (similar to the vertexwise effect shown in Figure 8F) and decreases in cortical volume in AC, HG and STG (3.9%, 4.5% and 2.4% respectively). All effect sizes for the ROI analysis are reported in Tables SI6–SI10 and significant ones are summarized in Table 3).

#### Grouping 2—Severe Tinnitus vs. Matched Controls

Most significant clusters for this subgroup analysis were for the effect of age and gender and were in a direction similar to the ones described in the whole-head analysis with all subjects (grouping 1). These effects will not be described in detail here (see Tables SI1–SI5). There were a few significant clusters showing an effect of tinnitus, tinnitus severity or hearing loss (described in the following section), and none in the whole-head analysis.

**Figure 9:**
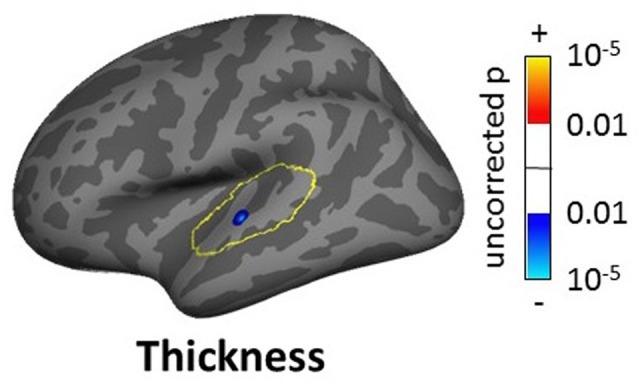
**Cluster showing a significant effect (*p* < 0.05 FWE-corrected) of tinnitus in left AC in the masked SBM analysis for the severe tinnitus grouping.** The yellow outlines depict the AC masks used to restrict the vertexwise analysis. The blue area corresponds to a negative effect (decrease in thickness for the severe tinnitus group vs. matched controls).

The masked voxelwise VBM analysis showed a 0.19 cm^3^ cluster of decreased white matter volume in the right MGN for the severe tinnitus subgroup compared to matched controls (Figure 7B; similar to the effect described above for all subjects and Figure 7A) and two clusters of decreasing gray matter volume with increasing hearing loss in the left IC (0.02 cm^3^; not shown) and the MGN bilaterally (1.22 cm^3^; not shown). The masked vertexwise SBM analysis showed a cluster of decreased cortical thickness for the severe tinnitus subgroup compared to matched controls at the junction of HG and the STG in the left hemisphere (0.85 cm^2^; Figure 9). There was also a cluster of increasing cortical thickness with increasing hearing loss in the right inferior parietal gyrus (1.47 cm^2^, not shown).

The ROI-averaged analysis showed no significant effect of tinnitus for either the VBM or SBM analyses.

#### Grouping 3—Tinnitus With Clinically Normal Hearing vs. Matched Controls

As for grouping 2, most significant clusters were for the effect of age or gender. There were however a number of clusters of significance for the effect of tinnitus, tinnitus severity and hearing loss, including a few in the whole-head analyses.

**Figure 10:**
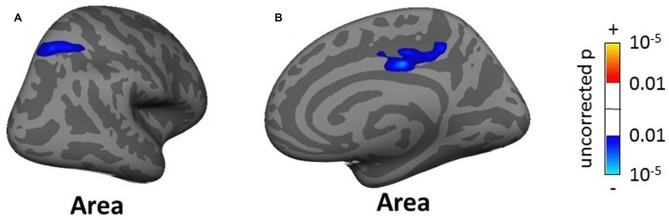
**Clusters showing a significant effect (*p* < 0.05 FWE-corrected) of tinnitus severity in right superior parietal gyrus (A) and right posterior cingulate cortex (B) in the whole-brain SBM analyses.** Blue areas correspond to a negative effect (decrease in cortical area with increasing tinnitus severity).

The whole-brain SBM analysis showed two clusters of decreasing cortical area with increasing tinnitus severity in the right superior parietal gyrus and pCC (total areas of clusters 12.17 cm^2^, Figure 10) and one cluster of increasing cortical volume with hearing loss in the right inferior parietal gyrus (4.55 cm^2^, not shown).

The masked VBM analysis showed a few small clusters of significance: one voxel in left HG and another in left CN showing an increase in white matter volume for the normal-hearing tinnitus group compared to matched controls, a 0.12 cm^3^ cluster in IC bilaterally showing increasing gray matter volume with hearing loss and two voxels (0.016 cm^3^) in the right AC (found using both the AC and STG masks) showing increasing white matter volume with hearing loss (not shown).

**Figure 11:**
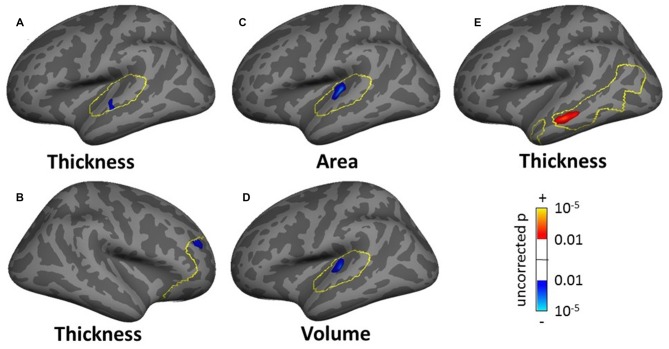
**Clusters showing a significant effect (*p* < 0.05 FWE-corrected) of tinnitus in left AC (A) and right rostromedial frontal cortex (B) of tinnitus severity in left HG (C,D) and left MTG (E) in the masked SBM analyses.** The yellow outlines depict the masks used to restrict the vertexwise analysis (AC for **A,C,D**; PFC for **B**; DMN for **E**). Blue areas correspond to a negative effect (decrease in thickness for the normal-hearing tinnitus group vs. matched controls in **(A,B)** decrease in area and volume with increasing tinnitus severity in **(C,D)** and the red areas to a positive effect (increasing thickness for increasing tinnitus severity in **(E)**.

The masked SBM analysis showed several clusters of significance for the effect of tinnitus and tinnitus severity: two clusters of decreased cortical thickness for the normal-hearing tinnitus group vs. matched controls in the left STG (1.17 cm^2^, Figure 11A, found both using the STG and AC masks) and in the right rostro-middle frontal gyrus (1.71 cm^2^, Figure 11B), one cluster of decreasing cortical area/volume with increasing tinnitus severity in left HG (3.18 cm^2^, Figures 11C,D found using the AC, HG and STG masks) and one cluster of increasing cortical thickness with increasing tinnitus severity in the left MTG (3.18 cm^2^, Figure 11E). There was also one cluster of increasing cortical thickness with hearing loss in the right rostro-middle frontal gyrus (1.81 cm^2^, not shown).

The ROI-averaged analysis showed no significant effect of tinnitus for either the VBM or SBM analyses.

## Discussion

Structural analysis of neuroanatomy offers a unique approach to unraveling the mystery of tinnitus. Different morphological techniques have unique strengths and limitations and results can vary depending on specific algorithms used to register or segment the brain and quantify changes in tissue type. Our study applied a range of these techniques to the same dataset—bringing novel insights into just how variable the findings from structural analysis of the brain can be.

While the large cohort of participants allowed us to control confounding effects of hearing loss and age, these two variables were correlated and so their independent effects cannot be isolated with any degree of precision. Controlling hearing loss and tinnitus severity, we found moderate changes in brain anatomy associated with tinnitus. Important and somewhat disappointing, many of these significant changes were different and some even contradicted findings from previous studies (see Adjamian et al., 2014).

### Tinnitus-related Changes—Comparison With Previous Findings

The results of both our VBM and SBM analyses reveal differences between tinnitus and non-tinnitus participants in both cortical and subcortical auditory structures, but only when the analysis was focused on these regions (masked voxel/vertexwise analyses or ROI analysis). Furthermore, as shown in Table 4, there was a limited overlap between the location and direction of our effects and those of previously published VBM and SBM studies.

**Table 4:** **Summary of main results of previous tinnitus VBM and SBM studies compared to present results (table adapted from Adjamian et al., 2014)**.

	Group differences		Modulations	
Brain structure	Decreases	Increases	HL	TIN
**Auditory gray matter**
Superior olivary complex	K		
Inferior colliculus	B	–	–	-
Medial geniculate body	FK	A	–	-
Heschl’s gyrus (A1)	***CG***K*K*	I	***CG***K	I
Superior temporal gyrus (A2)	*GK*	DF	DI	H*H*
**Non-auditory gray matter**
vmPFC/subcallosal gyrus	AE*G*H*H*	*(K)*	IJK	*K*
dmPFC	*G*H*H (K)*	D	DIJ	**-**
Nucleus accumbens	–	–	K	-
Anterior cingulate	*G*	D	D	-
Posterior cingulate	*G*	–	J	-
Hippocampus	BI	–	I	-
Insula	–	–	–	H*H*
Supramarginal gyrus	H	–	I	H*H*
Occipito-parietal cortex	–	I	I	-
Orbito-frontal cortex	F	–	–	-
Superior frontal gyrus	*(K)*	–	–	-
Middle frontal gyrus	—	*(K)*	–	-
Middle temporal gyrus	–	I*K*	–	-
Precuneus	–	–	–	*K*
Fusiform gyrus	–	–	*K*	-

*Present results are denoted by the letter K and are from grouping 1 only. Regular font letters denote VBM analyses and bold/italics letters SBM analyses. A = Mühlau et al. (2006); B = Landgrebe et al. (2009); C = Schneider et al. (2009); D = Husain et al. (2011); E = Leaver et al. (2011); F = Mahoney et al. (2011); G = Aldhafeeri et al. (2012); H = Leaver et al. (2012); I = Boyen et al. (2013); J = (Melcher et al. (2013); HL, Hearing loss; TIN, Tinnitus severity; vmPFC, ventromedial pre-frontal cortex; dmPFC, dorsomedial prefrontal Cortex*.

At the subcortical level, our VBM analysis showed an increase in gray matter concentration in the SOC and a reduction in white matter probability in the MGN (Figure 7A) for tinnitus participants. None of these effects have been reported before, although there are conflicting reports of changes in gray matter concentration in the medial geniculate body (MGB), with Mühlau et al. (2006) reporting an increase, and Mahoney et al. (2011) a decrease in tinnitus compared to controls. At the cortical level, we found small decreases in gray matter probability and/or thickness in right HG using both the VBM and SBM analyses (Figures 8C,D), as well as slightly larger decreases in cortical thickness in left AC (outside HG, Figure 8A). This is fairly consistent with previous SBM studies which reported a decrease in cortical thickness in right HG and STG bilaterally (Aldhafeeri et al., 2012) and a decrease in cortical volume in HG (Schneider et al., 2009). It should be borne in mind however that not all SBM studies have found this effect (see Leaver et al., 2012) and that VBM studies have tended to find increases rather than decreases in gray matter concentration in HG or STG in participants with tinnitus (Husain et al., 2011; Mahoney et al., 2011; Boyen et al., 2013).

The only two significant clusters of change related to tinnitus that we found in our whole-brain analysis were located outside sensory auditory structures: we found a decrease in cortical thickness for tinnitus participants in the left SFG and a decrease in cortical volume with tinnitus severity in the right precuneus (Figure 6). Whereas the effect in precuneus has not been reported before, the location of the effect in the SFG is very similar to that of the gray matter (thickness) reduction reported by Leaver et al. (2012) in dorsomedial PFC using both VBM and SBM, although theirs was on the right side. Aldhafeeri et al. (2012) also reported a general decrease in cortical thickness in the right PFC using SBM. In contrast, Husain et al. (2011) reported an increase in gray matter concentration in tinnitus participants using VBM.

When restricting the analysis to ROIs, we found a few additional effects of tinnitus in non-auditory structures (e.g., increase in cortical thickness with tinnitus severity in MTG in DMN mask). One area of particular interest is the vmPFC: in separate VBM or SBM studies, Mühlau et al. (2006), Leaver et al. (2011) and Leaver et al. (2012) found a decrease in gray matter concentration in tinnitus participants in the subcallosal region, which is included in the vmPFC. On the other hand, at least three other studies have failed to replicate these effects (Landgrebe et al., 2009; Husain et al., 2011; Melcher et al., 2013) even though some of them were specifically designed to do so. Here we find an effect that clearly contradicts previous studies, since our SBM analysis shows a general increase in cortical area in vmPFC for the tinnitus group (ROI analysis) as well as a more focused increase in cortical thickness with tinnitus severity in the right rostro medial frontal gyrus (in the vmPFC-masked vertexwise analysis, Figure 8F).

Finally, we note that many significant effects of tinnitus reported in earlier studies (in cingulate cortex, hippocampus, insula, supramarginal gyrus, occipito-parietal cortex; Landgrebe et al., 2009; Leaver et al., 2011; Mahoney et al., 2011; Aldhafeeri et al., 2012; Boyen et al., 2013; Husain and Schmidt, 2014) were not replicated here. Therefore, even though we did find effects that replicated previous studies, the overall picture is one of non-replicability and contradiction.

In this study, we used both VBM and SBM analysis techniques to assess gray/white matter concentration, and thickness, area, and volume of the gray matter, respectively. One might expect that any regions found in the gray matter VBM analysis would also appear in the SBM analysis (at least for the cortical volume measurement), since we applied these to the exact same data. However, we observed a proportionally larger and a higher number of significant clusters in SBM than VBM, with differing locations. There are several differences in the exact details of the VBM and SBM procedures that could explain these discrepancies. First, the nature of the measurement is different: VBM as implemented in SPM measures the probability that a certain voxel belongs to white or gray matter and this is modulated by the amplitude of the deformation necessary to register individual brains onto a common template. On the other hand, SBM as implemented in Freesurfer measures actual geometric properties of the cortical sheet (thickness, area and volume) at each vertex of each subject (modulated by the deformation fields for area and volume). Second, the registration procedure is different: SPM uses non-linear registration in volumetric space, whereas FreeSurfer uses spherical registration in surface space. Whereas in Freesurfer, deformation fields are necessarily limited to the cortical sheet, in SPM, they can occur in any direction in volume space. As a result, errors due to imperfect registration will be different in the two techniques. Finally, even though we used almost identical statistical models, there were at least two important differences: we did not correct for total/average brain thickness/area/volume in the SBM analysis and we used different types of FWE correction. The latter could explain why significant cluster sizes are proportionally larger in the SBM analysis: in the VBM analysis, the cluster size corresponds to the voxelwise-corrected *p*-value whereas in the SBM analysis, it corresponded to the uncorrected *p*-value (and is therefore larger). It is also likely that the cluster-based FWE correction used in Freesurfer is more sensitive to large clusters of relatively low significance, which could explain why more clusters were found overall in the SBM analysis.

### Reasons for Variability Between Studies

Tinnitus is a heterogeneous disorder, typically based on only a single criterion: the perception of a phantom sound. As yet there is no universally agreed separation of tinnitus patients into subgroups based on clinical and etiological factors. Tinnitus etiology, severity, hearing loss, comorbid medical conditions, age of onset, duration and laterality, among others, are factors which can effect brain morphology (Adjamian et al., 2014). Indeed, age and hearing loss seriously confound the interpretation of many results in this field. It is likely that the differences between participants across different studies, exaggerated by the lack of meaningful definitions of tinnitus subgroups, explain the reasons for diversity in findings.

A recent European-funded Cooperation in Science and Technology program (COST Action) for a Tinnitus research Network (TINNET^3^) aims to identify subtypes of tinnitus, and their neural correlates and thus develop an innovative hypothesis-driven treatment approaches. Until such time, future studies should attempt to collect as much information from participants as possible and attempt to recruit participants that are clinically and characteristically homogeneous as far as possible. Studies should ideally administer tinnitus questionnaires, depression questionnaires, measure audiograms at least up to 12 kHz, ascertain the duration, lateralization and cause of their tinnitus and basic demographic information. Participants should be matched on these characteristics as far as possible. Many of these variables were measured in the studies from which the data for the current analysis were obtained.

## Conclusion

Given the results of the present study, and in the context of previous discrepant findings, we conclude that it is not yet possible with any confidence to associate tinnitus with anatomical changes in specific parts of the brain. This is likely due to the heterogeneity of tinnitus characteristics, and the lack meaningful subtyping. Exploratory analyses might propose a subtyping classification which could then generate hypotheses for future testing. However, the more stringent the eligibility criteria for inclusion, the more challenging it will be to recruit sufficient number of participants in each subgroup for valid statistical inference.

## Author Contributions

PA and DRML conceived the study, wrote the discussion. PA and JD collected the data. TWA and JB analyzed the data and wrote the article. ARP and DAH supervised and provided guidance.

## Conflict of Interest Statement

The authors declare that the research was conducted in the absence of any commercial or financial relationships that could be construed as a potential conflict of interest.

## Footnotes

^1^http://www.fil.ion.ucl.ac.uk/spm/software/spm8/

^2^http://surfer.nmr.mgh.harvard.edu/

^3^http://tinnet.tinnitusresearch.net/
