# Supplementary material for: Neuroanatomical Alterations in Tinnitus Assessed with Magnetic Resonance Imaging
Source: Front Aging Neurosci. 2016 Sep 21;8:221. doi: 10.3389/fnagi.2016.00221 (PMC5030287; doi:10.3389/fnagi.2016.00221)
Supplement: Supplementary file 1 [file DataSheet_1.docx]

**Supplementary information – Detailed Results**

**Title: “Neuroanatomical alterations in tinnitus assessed with magnetic resonance imaging”**

**Authors:** Thomas Allan, Julien Besle, Dave R.M. Langers, Jeff Davies, Deborah A. Hall, Alan R. Palmer, and Peyman Adjamian

**Corresponding Author:**

Dr Peyman Adjamian

MRC Institute of Hearing Research, University of Nottingham, University Park, Nottingham NG7 2RD, United Kingdom

| **Grey Matter** | | |  |  |  |  |  |  |  |  |  |  |  |  |  |
| --- | --- | --- | --- | --- | --- | --- | --- | --- | --- | --- | --- | --- | --- | --- | --- |
| (VBM) | | **Contrast** |  |  | **WH** | **AC** | **CN** | **DMN** | **HG** | **IC** | **MGN** | **NAc** | **PFC** | **SOC** | **STG** |
| **Grouping 1** | *(All TIs vs all COs)* | TI vs CO | P |  | - | - | - | - | - | - | - | - | - | - | - |
|  |  |  | N |  | - | - | - | - | 1 | - | - | - | - | - | - |
|  |  | TI severity | P |  | - | - | - | - | - | - | - | - | - | - | - |
|  |  |  | N |  | - | - | - | 7 | - | - | - | - | - | - | - |
|  |  | HL | P |  | - | - | - | - | - | - | - | - | - | - | - |
|  |  |  | N |  | - | - | - | - | - | - | - | - | - | - | - |
|  |  | Age | P |  | 10234 | 30 | 109 | 47 | 39 | 162 | 294 | 7 | 13 | 52 | 76 |
|  |  |  | N |  | 25 | 6 | - | 9 | - | - | - | 15 | 74 | - | 2 |
|  |  | Gender | P |  | 1737 | 19 | - | 12 | - | - | - | - | - | - | 20 |
|  |  |  | N |  | 346 | - | - | - | 6 | - | - | - | 6 | - | - |
| **Grouping 2** | *(severe TI vs matched controls)* | TI vs CO | P |  | - | - | - | - | - | - | - | - | - | - | - |
|  |  |  | N |  | - | - | - | - | - | - | - | - | - | - | - |
|  |  | TI severity | P |  | - | - | - | - | - | - | - | - | - | - | - |
|  |  |  | N |  | - | - | - | - | - | - | - | - | - | - | - |
|  |  | HL | P |  | - | - | - | - | - | - | - | - | - | - | - |
|  |  |  | N |  | - | - | - | - | - | 2 | 153 | - | - | - | - |
|  |  | Age | P |  | 70 | - | 5 | - | - | 146 | 410 | - | - | 29 | - |
|  |  |  | N |  | - | 2 | - | - | - | - | - | - | - | - | 1 |
|  |  | Gender | P |  | - | - | - | - | - | - | - | - | - | - | - |
|  |  |  | N |  | - | - | - | - | - | - | - | - | - | - | - |
| **Grouping 3** | *(TI normal hearing vs matched controls)* | TI vs CO | P |  | - | - | - | - | - | - | - | - | - | - | - |
|  |  |  | N |  | - | - | - | - | - | - | - | - | - | - | - |
|  |  | TI severity | P |  | - | - | - | - | - | - | - | - | - | - | - |
|  |  |  | N |  | - | - | - | - | - | - | - | - | - | - | - |
|  |  | HL | P |  | - | - | - | - | - | - | - | - | - | - | - |
|  |  |  | N |  | - | - | - | - | - | 15 | - | - | - | - | - |
|  |  | Age | P |  | - | - | - | - | 4 | 69 | 62 | - | - | - | - |
|  |  |  | N |  | - | 7 | - | 4 | - | - | - | - | 1 | - | 4 |
|  |  | Gender | P |  | - | - | - | - | - | - | - | - | - | - | - |
|  |  |  | N |  | - | - | - | - | - | - | 7 | - | - | - | - |

**Table SI 1**. Number of voxels showing a (FWE-corrected) significant effect of each of the 5 tested contrasts on grey matter volume, for each grouping and mask, in the VBM analysis. Numbers are broken down into positive (P) or negative (N) slope effects (Positive = increase in volume for TI vs CO, with TI severity, with hearing loss, with age and for Males vs Female). Dashes indicate an absence of significant voxels. TI=Tinnitus, CO=Controls, WH=Whole head. For other abbreviations, see Table 1 in main article.

| **White Matter** | | |  |  |  |  |  |  |  |  |  |  |  |  |  |
| --- | --- | --- | --- | --- | --- | --- | --- | --- | --- | --- | --- | --- | --- | --- | --- |
| (VBM) | | **Contrast** |  |  | **WH** | **AC** | **CN** | **DMN** | **HG** | **IC** | **MGN** | **NAc** | **PFC** | **SOC** | **STG** |
| **Grouping 1** | *(All TIs vs all COs)* | TI vs CO | P |  | - | - | - | - | - | - | - | - | - | - | - |
|  |  |  | N |  | - | - | - | - | - | - | 63 | - | - | - | - |
|  |  | TI severity | P |  | - | - | - | - | - | - | - | - | - | - | - |
|  |  |  | N |  | - | - | - | - | - | - | - | - | - | - | - |
|  |  | HL | P |  | - | - | - | - | - | - | - | 13 | - | - | - |
|  |  |  | N |  | - | 18 | - | - | 66 | - | - | - | 7 | - | 50 |
|  |  | Age | P |  | 70 | 8 | - | 5 | 102 | - | - | 19 | 19 | 22 | 3 |
|  |  |  | N |  | 4335 | - | - | 772 | - | - | 376 | - | 675 | - | - |
|  |  | Gender | P |  | 88 | 25 | - | 1 | 7 | - | - | - | 79 | - | 13 |
|  |  |  | N |  | 163 | 21 | 162 | 0 | 42 | 18 | 301 | 27 | 20 | 137 | - |
| **Grouping 2** | *(severe TI vs matched controls)* | TI vs CO | P |  | - | - | - | - | - | - | - | - | - | - | - |
|  |  |  | N |  | - | - | - | - | - | - | 24 | - | - | - | - |
|  |  | TI severity | P |  | - | - | - | - | - | - | - | - | - | - | - |
|  |  |  | N |  | - | - | - | - | - | - | - | - | - | - | - |
|  |  | HL | P |  | - | - | - | - | - | - | - | - | - | - | - |
|  |  |  | N |  | - | - | - | - | - | - | - | - | - | - | - |
|  |  | Age | P |  | - | - | - | - | - | - | - | 7 | - | - | - |
|  |  |  | N |  | - | - | - | - | - | - | - | - | 1 | - | - |
|  |  | Gender | P |  | - | - | - | - | - | - | - | - | - | - | 1 |
|  |  |  | N |  | - | - | - | - | - | - | 50 | 2 | - | - | - |
| **Grouping 3** | *(TI normal hearing vs matched controls)* | TI vs CO | P |  | - | - | - | - | 1 | - | - | - | - | - | - |
|  |  |  | N |  | - | - | - | - | - | - | - | - | - | - | - |
|  |  | TI severity | P |  | - | - | 1 | - | - | - | - | - | - | - | - |
|  |  |  | N |  | - | - | - | - | - | - | - | - | - | - | - |
|  |  | HL | P |  | - | 2 | - | - | - | - | - | - | - | - | 2 |
|  |  |  | N |  | - | - | - | - | - | - | - | - | - | - | - |
|  |  | Age | P |  | - | - | - | - | - | - | - | - | - | - | - |
|  |  |  | N |  | - | - | - | - | - | - | - | - | - | - | - |
|  |  | Gender | P |  | - | - | - | - | - | - | - | - | - | - | 5 |
|  |  |  | N |  | - | - | 6 | - | - | - | - | - | - | - | - |

**Table SI 2.** Number of voxels showing a (FWE-corrected) significant effect of each of the 5 tested contrasts on white matter volume, for each grouping and mask, in the VBM analysis. Numbers are broken down into positive (P) or negative (N) slope effects (Positive = increase in volume for TI vs CO, with TI severity, with hearing loss, with age and for Males vs Female). Dashes indicate an absence of significant voxels. TI=Tinnitus, CO=Controls, WH=Whole head. For other abbreviations, see Table 1 in main article.

**Table SI 3.** Total surface area of significant clusters showing a (FWE-corrected) significant effect of each of the 5 tested contrasts on cortical thickness, for each grouping and mask, in the SBM analysis (in mm^2^). Numbers are broken down into positive (P) or negative (N) slope effects (Positive = increase in volume for TI vs CO, with TI severity, with hearing loss, with age and for Males vs Female) and left and right hemispheres. Dashes indicate an absence of significant clusters. TI=Tinnitus, CO=Controls, WH=Whole head. For other abbreviations, see Table 1 in main article.

**Table SI 4.** Total surface area of significant clusters showing a (FWE-corrected) significant effect of each of the 5 tested contrasts on cortical area, for each grouping and mask, in the SBM analysis (in mm^2^). Numbers are broken down into positive (P) or negative (N) slope effects (Positive = increase in volume for TI vs CO, with TI severity, with hearing loss, with age and for Males vs Female) and left and right hemispheres. Dashes indicate an absence of significant clusters. TI=Tinnitus, CO=Controls, WH=Whole head. For other abbreviations, see Table 1 in main article.

**Table SI 5.** Total surface area of significant clusters showing a (FWE-corrected) significant effect of each of the 5 tested contrasts on cortical volume, for each grouping and mask, in the SBM analysis (in mm^2^). Numbers are broken down into positive (P) or negative (N) slope effects (Positive = increase in volume for TI vs CO, with TI severity, with hearing loss, with age and for Males vs Female) and left and right hemispheres. Dashes indicate an absence of significant clusters. TI=Tinnitus, CO=Controls, WH=Whole head. For other abbreviations, see Table 1 in main article.

| **(VBM)** | | **Tinnitus** | | | | **Controls** | | | | | **Tinnitus** | | | | | | | **Controls** | | | | | | **Tinnitus** | | | | | | **Controls** | | |  |
| --- | --- | --- | --- | --- | --- | --- | --- | --- | --- | --- | --- | --- | --- | --- | --- | --- | --- | --- | --- | --- | --- | --- | --- | --- | --- | --- | --- | --- | --- | --- | --- | --- | --- |
| **Network** | | **Mean** | | **Std** | | **Mean** | | **Std** | | | **Mean** | | | | **Std** | | | **Mean** | | | **Std** | | | **Mean** | | | | **Std** | | **Mean** | | **Std** |  |
| **AC** | | 0.3624 | | 0.0409 | | 0.3602 | | 0.0376 | | | 0.3625 | | | | 0.0422 | | | 0.3650 | | | 0.0460 | | | 0.3624 | | | | 0.0320 | | 0.3627 | | 0.0382 |  |
| **CN** | | 0.1275 | | 0.0157 | | 0.1239 | | 0.0130 | | | 0.1249 | | | | 0.0173 | | | 0.1215 | | | 0.0104 | | | 0.1203 | | | | 0.0125 | | 0.1193 | | 0.0107 |  |
| **DMN** | | 0.3943 | | 0.0410 | | 0.3892 | | 0.0427 | | | 0.3907 | | | | 0.0442 | | | 0.3947 | | | 0.0552 | | | 0.3924 | | | | 0.0344 | | 0.3872 | | 0.0421 |  |
| **H** | | 0.3758 | | 0.0551 | | 0.3714 | | 0.0420 | | | 0.3802 | | | | 0.0587 | | | 0.3755 | | | 0.0504 | | | 0.3792 | | | | 0.0448 | | 0.3742 | | 0.0474 |  |
| **IC** | | 0.1891 | | 0.0198 | | 0.1861 | | 0.0195 | | | 0.1810 | | | | 0.0216 | | | 0.1840 | | | 0.0195 | | | 0.1801 | | | | 0.0137 | | 0.1864 | | 0.0181 |  |
| **MGN** | | 0.1632 | | 0.0153 | | 0.1605 | | 0.0158 | | | 0.1593 | | | | 0.0170 | | | 0.1589 | | | 0.0154 | | | 0.1611 | | | | 0.0129 | | 0.1594 | | 0.0160 |  |
| **NAc** | | 0.4703 | | 0.0480 | | 0.4647 | | 0.0512 | | | 0.4654 | | | | 0.0397 | | | 0.4667 | | | 0.0593 | | | 0.4728 | | | | 0.0473 | | 0.4552 | | 0.0469 |  |
| **PFC** | | 0.3655 | | 0.0418 | | 0.3567 | | 0.0415 | | | 0.3614 | | | | 0.0422 | | | 0.3611 | | | 0.0547 | | | 0.3674 | | | | 0.0366 | | 0.3563 | | 0.0423 |  |
| **SOC** | | 0.0696 | | 0.0114 | | 0.0660 | | 0.0083 | | | 0.0664 | | | | 0.0109 | | | 0.0657 | | | 0.0079 | | | 0.0648 | | | | 0.0064 | | 0.0634 | | 0.0069 |  |
| **STG** | | 0.3809 | | 0.0389 | | 0.3775 | | 0.0389 | | | 0.3805 | | | | 0.0376 | | | 0.3824 | | | 0.0490 | | | 0.3795 | | | | 0.0291 | | 0.3763 | | 0.0383 |  |
|  | |  | |  | |  | |  | | |  | | | |  | | |  | | |  | | |  | | | |  | |  | |  |  |
|  |  | |  | |  | |  | | |  | | |  | | |  | | |  | | |  | | |  | |  |  |  |  |  |  |  |
|  |  | |  | | **% change** | |  | |  | | |  | |  | | |  | | |  | | |  | | |  |  |  |  |  |  |  |  |
|  | | **SOC** | |  | | 0.0036 | |  | | | 5.31 | | | |  | | |  | | |  | | |  | | | |  |  | |  |  |  |

**Table SI 6**. The effect size of each group and mask showing the mean volume of grey matter and associated standard deviation (in cm^3^/voxel) for each group for the VBM analysis. For abbreviations, see Table 1 in main article.

|  |  |  |  |  |  |  |  |  |  |  |  |  |  |
| --- | --- | --- | --- | --- | --- | --- | --- | --- | --- | --- | --- | --- | --- |
| **(VBM)** | | **Tinnitus** | | **Controls** | | **Tinnitus** | | **Controls** | | **Tinnitus** | | **Controls** | |
| **Network** | | **Mean** | **Std** | **Mean** | **Std** | **Mean** | **Std** | **Mean** | **Std** | **Mean** | **Std** | **Mean** | **Std** |
|  | |  |  |  |  |  |  |  |  |  |  |  |  |
| **AC** | | 0.1806 | 0.0198 | 0.1797 | 0.0210 | 0.1780 | 0.0179 | 0.1801 | 0.0230 | 0.1793 | 0.0181 | 0.1777 | 0.0238 |
|  | |  |  |  |  |  |  |  |  |  |  |  |  |
| **CN** | | 0.4916 | 0.0545 | 0.4888 | 0.0486 | 0.4861 | 0.0569 | 0.4892 | 0.0508 | 0.4909 | 0.0459 | 0.4883 | 0.0347 |
|  | |  |  |  |  |  |  |  |  |  |  |  |  |
| **DMN** | | 0.2439 | 0.0271 | 0.2379 | 0.0325 | 0.2362 | 0.0248 | 0.2410 | 0.0409 | 0.2399 | 0.0238 | 0.2371 | 0.0336 |
|  | |  |  |  |  |  |  |  |  |  |  |  |  |
| **H** | | 0.3257 | 0.0367 | 0.3232 | 0.0360 | 0.3222 | 0.0352 | 0.3195 | 0.0374 | 0.3241 | 0.0361 | 0.3223 | 0.0439 |
|  | |  |  |  |  |  |  |  |  |  |  |  |  |
| **IC** | | 0.3703 | 0.0436 | 0.3739 | 0.0372 | 0.3621 | 0.0460 | 0.3786 | 0.0430 | 0.3693 | 0.0397 | 0.3737 | 0.0287 |
|  | |  |  |  |  |  |  |  |  |  |  |  |  |
| **MGN** | | 0.5640 | 0.0582 | 0.5676 | 0.0540 | 0.5497 | 0.0538 | 0.5745 | 0.0629 | 0.5666 | 0.0528 | 0.5678 | 0.0480 |
|  | |  |  |  |  |  |  |  |  |  |  |  |  |
| **NAc** | | 0.2077 | 0.0209 | 0.2053 | 0.0180 | 0.2018 | 0.0183 | 0.2054 | 0.0209 | 0.2018 | 0.0150 | 0.2030 | 0.0157 |
|  | |  |  |  |  |  |  |  |  |  |  |  |  |
| **PFC** | | 0.1672 | 0.0210 | 0.1627 | 0.0212 | 0.1616 | 0.0175 | 0.1650 | 0.0265 | 0.1667 | 0.0202 | 0.1635 | 0.0227 |
|  | |  |  |  |  |  |  |  |  |  |  |  |  |
| **SOC** | | 0.5808 | 0.0713 | 0.5781 | 0.0659 | 0.5724 | 0.0695 | 0.5756 | 0.0708 | 0.5837 | 0.0641 | 0.5807 | 0.0473 |
|  | |  |  |  |  |  |  |  |  |  |  |  |  |
| **STG** | | 0.1790 | 0.0198 | 0.1784 | 0.0222 | 0.1764 | 0.0180 | 0.1807 | 0.0250 | 0.1770 | 0.0176 | 0.1775 | 0.0255 |

**Table SI 7**. The effect size of each group and mask showing the mean volume of white matter and associated standard deviation (in cm^3^/voxel) for each group for the VBM analysis

| **Thickness** | | |  |  |  |  |  |  |  |  |  |  |  |  |  |  |  |  |  |  |  |  |  |  |  |  |  |  |  |
| --- | --- | --- | --- | --- | --- | --- | --- | --- | --- | --- | --- | --- | --- | --- | --- | --- | --- | --- | --- | --- | --- | --- | --- | --- | --- | --- | --- | --- | --- |
| **(SBM)** | |  | **Tinnitus** | | | | **Controls** | | | | **Tinnitus** | | | | **Controls** | | | | | | | **Tinnitus** | | | | | **Controls** | | |
| **Network** | |  | **Mean** | | **Std** | | **Mean** | | **Std** | | **Mean** | | **Std** | | **Mean** | | | **Std** | | | | **Mean** | | | **Std** | | **Mean** | **Std** | |
|  | |  |  | |  | |  | |  | |  | |  | |  | | |  | | | |  | | |  | |  |  | |
| **AC** | |  | 2.47 | | 0.14 | | 2.52 | | 0.13 | | 2.53 | | 0.17 | | 2.58 | | | 0.09 | | | | 2.57 | | | 0.18 | | 2.56 | 0.09 | |
|  | |  |  | |  | |  | |  | |  | |  | |  | | |  | | | |  | | |  | |  |  | |
| **DMN** | |  | 2.55 | | 0.10 | | 2.56 | | 0.11 | | 2.58 | | 0.11 | | 2.61 | | | 0.12 | | | | 2.59 | | | 0.12 | | 2.58 | 0.12 | |
|  | |  |  | |  | |  | |  | |  | |  | |  | | |  | | | |  | | |  | |  |  | |
| **H** | |  | 2.24 | | 0.16 | | 2.29 | | 0.13 | | 2.29 | | 0.20 | | 2.35 | | | 0.08 | | | | 2.33 | | | 0.20 | | 2.31 | 0.08 | |
|  | |  |  | |  | |  | |  | |  | |  | |  | | |  | | | |  | | |  | |  |  | |
| **PFC** | |  | 2.40 | | 0.10 | | 2.41 | | 0.11 | | 2.44 | | 0.09 | | 2.41 | | | 0.11 | | | | 2.42 | | | 0.13 | | 2.43 | 0.08 | |
|  | |  |  | |  | |  | |  | |  | |  | |  | | |  | | | |  | | |  | |  |  | |
| **STG** | |  | 2.70 | | 0.14 | | 2.74 | | 0.14 | | 2.76 | | 0.17 | | 2.80 | | | 0.11 | | | | 2.79 | | | 0.16 | | 2.77 | 0.10 | |
|  | |  |  | |  | |  | |  | |  | |  | |  | | |  | | | |  | | |  | |  |  | |
|  |  | | |  | |  | |  | |  | |  | |  | | |  | | |  | | |  | | |  |  |  |  |
| **All subjects** | | |  | |  | |  | | **% change** | |  |  | |  | |  | | |  | |  | | |  |  |  |  |  |  |
|  | |  | **AC** | |  | | -0.05 | |  | | -2.07 | |  | |  | | |  | | | |  | | |  | |  |  |  |

**Table SI 8.** The effect size of each group and mask showing the mean thickness of grey matter and the associated standard deviation (in mm) for each group for the SBM ROI analysis.

| **Area** | | |  |  |  |  |  |  |  |  |  |  |  |  |  |  |  |  |  |  |  |  |  |  |  |  |  |  |  |  |  |  |  |  |  |  |  |  |  |
| --- | --- | --- | --- | --- | --- | --- | --- | --- | --- | --- | --- | --- | --- | --- | --- | --- | --- | --- | --- | --- | --- | --- | --- | --- | --- | --- | --- | --- | --- | --- | --- | --- | --- | --- | --- | --- | --- | --- | --- |
| **(SBM)** | | | **Tinnitus** | | | | | | | **Controls** | | | | | | | | | **Tinnitus** | | | | | | | | | | **Controls** | | | | | | **Tinnitus** | | | **Controls** | |
| **Network** | | | **Mean** | | | | **Std** | | | **Mean** | | | | **Std** | | | | | **Mean** | | | | **Std** | | | | | | **Mean** | | | | **Std** | | **Mean** | | **Std** | **Mean** | **Std** |
|  | | |  | | | |  | | |  | | | |  | | | | |  | | | |  | | | | | |  | | | |  | |  | |  |  |  |
| **AC** | | | 3220 | | | | 196 | | | 3288 | | | | 153 | | | | | 3307 | | | | 182 | | | | | | 3288 | | | | 155 | | 3272 | | 175 | 3306 | 164 |
|  | | |  | | | |  | | |  | | | |  | | | | |  | | | |  | | | | | |  | | | |  | |  | |  |  |  |
| **DMN** | | | 10632 | | | | 499 | | | 10498 | | | | 474 | | | | | 10624 | | | | 580 | | | | | | 10701 | | | | 507 | | 10648 | | 598 | 10479 | 606 |
|  | | |  | | | |  | | |  | | | |  | | | | |  | | | |  | | | | | |  | | | |  | |  | |  |  |  |
| **H** | | | 1004 | | | | 112 | | | 1026 | | | | 96 | | | | | 1031 | | | | 104 | | | | | | 1017 | | | | 110 | | 1011 | | 95 | 1012 | 68 |
|  | | |  | | | |  | | |  | | | |  | | | | |  | | | |  | | | | | |  | | | |  | |  | |  |  |  |
| **PFC** | | | 15667 | | | | 594 | | | 15464 | | | | 535 | | | | | 15609 | | | | 674 | | | | | | 15561 | | | | 455 | | 15875 | | 747 | 15446 | 603 |
|  | | |  | | | |  | | |  | | | |  | | | | |  | | | |  | | | | | |  | | | |  | |  | |  |  |  |
| **STG** | | | 5404 | | | | 275 | | | 5464 | | | | 236 | | | | | 5546 | | | | 225 | | | | | | 5498 | | | | 254 | | 5462 | | 256 | 5486 | 209 |
|  | | |  | | | |  | | |  | | | |  | | | | |  | | | |  | | | | | |  | | | |  | |  | |  |  |  |
|  |  | | | |  | | | |  | | |  |  | | | | |  | | |  | | | | |  | |  | | | |  |  |  |  |  |  |  |  |
|  | |  | | | |  | |  | | | **% change** | | | |  |  | | | |  | | | | |  | | | | | |  |  |  |  |  |  |  |  |  |
|  | | | **AC** | | | |  | | | -68 | | | |  | | | -2.08 | | | | |  | |  | | |  | | |  | | | |  | |  |  |  |  |
|  | | | **PFC** | | | |  | | | 203 | | | |  | | | 1.31 | | | | |  | |  | | |  | | |  | | | |  | |  |  |  |  |

**Table SI 9.** The effect size of each group and mask showing the mean area of grey matter and the associated standard deviation (in mm^2^) for each group for the SBM analysis.

| **Volume** | |  |  |  |  |  |  |  |  |  |  |  |  |  |  |  |  |  |  |  |  |  |  |  |  |  |  |  |  |  |  |  |  |  |
| --- | --- | --- | --- | --- | --- | --- | --- | --- | --- | --- | --- | --- | --- | --- | --- | --- | --- | --- | --- | --- | --- | --- | --- | --- | --- | --- | --- | --- | --- | --- | --- | --- | --- | --- |
| **(SBM)** | | **Tinnitus** | | | | | | **Controls** | | | | | | | | | **Tinnitus** | | | | | | | | **Controls** | | | | | | **Tinnitus** | | **Controls** | |
| **Network** | | **Mean** | | | **Std** | | | **Mean** | | | | **Std** | | | | | **Mean** | | | | **Std** | | | | **Mean** | | | **Std** | | | **Mean** | **Std** | **Mean** | **Std** |
|  | |  | | |  | | |  | | | |  | | | | |  | | | |  | | | |  | | |  | | |  |  |  |  |
| **AC** | | 8840 | | | 641 | | | 9194 | | | | 494 | | | | | 9122 | | | | 572 | | | | 9256 | | | 503 | | | 9131 | 530 | 9247 | 509 |
|  | |  | | |  | | |  | | | |  | | | | |  | | | |  | | | |  | | |  | | |  |  |  |  |
| **DMN** | | 24111 | | | 1200 | | | 23858 | | | | 1303 | | | | | 24042 | | | | 1512 | | | | 24340 | | | 1485 | | | 24156 | 1568 | 23748 | 1861 |
|  | |  | | |  | | |  | | | |  | | | | |  | | | |  | | | |  | | |  | | |  |  |  |  |
| **H** | | 1662 | | | 199 | | | 1740 | | | | 167 | | | | | 1694 | | | | 182 | | | | 1756 | | | 220 | | | 1692 | 167 | 1710 | 149 |
|  | |  | | |  | | |  | | | |  | | | | |  | | | |  | | | |  | | |  | | |  |  |  |  |
| **PFC** | | 33982 | | | 1353 | | | 33558 | | | | 1219 | | | | | 34152 | | | | 1460 | | | | 33353 | | | 1053 | | | 34283 | 1506 | 33265 | 1307 |
|  | |  | | |  | | |  | | | |  | | | | |  | | | |  | | | |  | | |  | | |  |  |  |  |
| **STG** | | 14073 | | | 890 | | | 14414 | | | | 743 | | | | | 14532 | | | | 696 | | | | 14555 | | | 721 | | | 14342 | 789 | 14425 | 577 |
|  | |  | | |  | | |  | | | |  | | | | |  | | | |  | | | |  | | |  | | |  |  |  |  |
|  |  | | |  | | |  | | |  |  | | | | |  | | |  | | | |  |  | | |  | | |  |  |  |  |  |
|  |  | | |  | |  | | | **% change** | | | |  |  | | | |  | | | |  | | |  | | |  | | |  |  |  |  |
|  | | **AC** | | |  | | | -354 | | | |  | | | -3.93 | | | | |  |  | | | |  |  | | |  | |  |  |  |  |
|  | | **H** | | |  | | | -77 | | | |  | | | -4.54 | | | | |  |  | | | |  |  | | |  | |  |  |  |  |
|  | | **STG** | | |  | | | -341 | | | |  | | | -2.39 | | | | |  |  | | | |  |  | | |  | |  |  |  |  |

**Table SI 10.** The effect size of each group and mask showing the mean volume of grey matter and the associated standard deviation (in mm^3^) for each group for the SBM analysis.
